# The roles of Toll-like receptor 4 in the pathogenesis of pathogen-associated biliary fibrosis caused by *Clonorchis sinensis*

**DOI:** 10.1038/s41598-017-04018-8

**Published:** 2017-06-20

**Authors:** Chao Yan, Bo Li, Fang Fan, Ying Du, Rui Ma, Xiao-Dan Cheng, Xiang-Yang Li, Bo Zhang, Qian Yu, Yu-Gang Wang, Ren-Xian Tang, Kui-Yang Zheng

**Affiliations:** 1 Jiangsu Key Laboratory of Immunity and Metabolism, Department of Pathogenic Biology and Immunology, Laboratory of Infection and Immunity, Xuzhou Medical University, Xuzhou, 221004 Jiangsu Province People’s Republic of China; 2 The Second Affiliated Hospital of Nanjing Medical University, Nuclear Medicine Department, Nanjing, 210011 Jiangsu Province People’s Republic of China

## Abstract

Pathogen-associated biliary fibrosis (PABF) is a type of liver fibrosis characterized by injuries of cholangiocytes and extra cellular matrix (ECM) deposition around bile ducts caused by various bacteria, fungi, virus and parasites. Recent studies show that TLR4 plays an important role in several other types of liver fibrosis, but the mechanism of TLR4 in PABF is yet really unclear. In the present study, a PABF mouse model was established by a trematode infection-*Clonorchis sinensis* which dwells in the bile ducts and causes severe biliary fibrosis of mice. The results showed that the levels of collagen depositions, α-SMA and hydroxyproline (Hyp) contents in TLR4^*mut*^ mice infected by *C*. *sinensis* were significantly lower than in those of TLR4^*wild*^ ones. Furthermore, we found that the activation of TGF-β signaling was impaired in the TLR4^*mut*^ mice, compared with wild mice when they were challenged to the same dose of *C*. *sinensis* metacercariae. Moreover, the mice with TLR4 mutation showed a decreased activation of hepatic stellate cells indicated by the expression of α-SMA, when compared with TLR4^*wild*^ mice. These data demonstrate that TLR4 contributes to PABF caused by *C*. *sinensis* and TLR4 signaling may be a potential medical target for treatment of PABF.

## Introduction

Infections with pathogens such as bacteria, virus, fungi and parasites can cause biliary fibrosis, which is a chronic liver disease resulted from injuries to cholangiocytes and characterized by accumulation of extra cellular matrix (ECM) around bile ducts^1–5^. It can progress to biliary cirrhosis which is the leading course of liver failure and contribute to cholangiocarcinoma due to the lack of effective clinic therapies^6^. However, the pathogenesis of the pathogen-associated biliary fibrosis (PABF) is poorly understood as it may differ from other types of hepatic fibrosis^3, 7^.

*Clonorchis sinensis*, a food borne parasite is prevalent in the part of eastern Asia including China, Korea and Vietnam^8, 9^. And it is estimated that approximately 15 million people get infected via ingestion of raw or undercooked fish meat containing metacercariae of *C*. *sinensis* ^9, 10^. The adult worm dwells in the bile ducts of definitive hosts (such as humans, dogs and cats) and causes cholangitis, cholestasis, cholelithiasis and even cholangiocarcinoma^9^. Notably, chronic infection with *C*. *sinensis* can progress to biliary fibrosis accompanied by the portal/periportal infiltration of inflammatory cells such as T lymphocyte cells, macrophage cells and neutrophils, and the interaction of these cells may contribute to the process of PABF^11, 12^.

A variety of cells are thought to contribute to the production of ECM during biliary fibrosis, including hepatic stellate cells (HSCs), hepatic progenitor cells, periportal/portal fibroblasts and other mesenchymal cells via type 2 EMT^3, 13–15^. However, the activated HSCs with high expressed α-smooth muscle actin (α-SMA) are considered as the most potent effective cells that differentiate into ECM-producing myofibroblasts^16^. Therefore, the activation of HSCs is a central event during hepatic fibrosis, which is orchestrated by a complex regulatory network of cytokines and reactive oxygen species^17, 18^. Although many cytokines are involved in the process of hepatic fibrosis, transforming growth factor-β1 (TGF-β1) is a major pro-fibrogenic cytokine that orchestrates the fibrotic responses and plays a predominant role in the activation of HSCs, as well as promotion of ECM production through TGF-β/Smads signaling pathway^19, 20^.

Toll-like receptors as one of the most important pattern recognition receptors for foreign pathogen-associated molecular patterns (PAMPs) contribute to host defenses by innate immunity as well as control the adaptive immune responses of hosts^21^. Recently, it has been demonstrated that TLR4 involves in the pathogenesis of fibrosis including hepatic fibrosis and TLR4 is a key regulator of HSC activation^22^. The activated TLR4 signaling can orchestrate a cross talk between BM-derived cells and endogenous liver cells to promote the release of TGF-β and sensitization of HSCs to TGF-β for HSC activation^22–24^. However, the direct contribution of TLR4 to pathogen-associated biliary fibrosis has not been addressed. Our previous studies showed that TLR4 was highly expressed in the activated HSC cells and myofibroblasts during *C*. *sinensis*-caused biliary fibrosis^11^, but the exact role of TLR4 in the pathological process of PABF caused by *C*. *sinensis* still remains unknown. Therefore, in the present study, the aim of the present study was to elucidate the mechanisms by which TLR4 orchestrates PABF caused by *C*. *sinensis in vivo*. The results of the present study will contribute to a better understanding of pathogen-associated biliary fibrogenesis due to the damage of cholangiocytes caused by the pathogen.

## Materials and Methods

### Ethics statement

The mice were housed in an air-conditioned room at 24 °C with a 12 h dark/light cycle and were fed *ad libitum* under specific pathogen-free conditions. All animal experimental protocols and procedures were reviewed and approved by the Animal Care and Use Committee of Xuzhou Medical University (Permit Number 2013-AN-65) according to the guidelines of National Laboratory Animal Ethics Committee of China.

### Animals and parasites

Metacercariae of *C*. *sinensis* from *Pseudorasbora parva* were obtained by digesting fish with a pepsin-HCl (0.6%) artificial gastric juice as described elsewhere^25^.

Age-matched 6–8 week old TLR4^*wild*^ (C3H/HeN) and TLR4^*mut*^ (C3H/HeJ) mice were introduced from the Jackson Laboratory (Bar Harbor, ME) and maintained in model animal research center of Xuzhou Medical University (Xuhzou, Jiangsu, China). Mice were administrated 45 metacercariae by intragastic intubation. The mice were sacrificed at four weeks post infection. The tissue and serum from each mouse were collected and stored in −80 °C for further use.

### Masson staining

Liver specimens were fixed with 4% paraformaldehyde for 24 h, embedded in paraffin and cut into 3~4-μm serial thick sections for Masson staining according to the manufacturer’s instructions (Jiancheng, Nanjing, Jiangsu, China). After sealing the slides with neutral gum, the stained tissue slices were microscopically examined at 40 × magnification. Subsequently, color images in five randomly chosen microscopic fields of each slide were captured and analyzed by a medical image software program (Image-Pro Plus, Media Cybernetics, USA) to semi-quantitatively determine optical density (IOD) i.e. collagen fiber area over section area by two independed pathologists.

### Immunohistochemistry

The liver tissue from each strain mice was fixed in 4% formalin solution and embedded in paraffin. The paraffin-embedded liver was sectioned (3~4 μm thickness). For immunohistochemically staining of *α*-smooth muscle actin (*α*-SMA), liver tissue were deparaffinized, hydrated, and heated in antibody specific retrieval buffer (citric acid buffer) at 95 °C for 8 min and then treated with goat serum for 30 min. The slides were then incubated overnight with primary antibodies α-SMA (1:200, Ucallm biotech Co., Ltd, Wuxi, China). After washing with tap water, DAB (ZSGB–BIO, Beijing, China) as a substrate was added. The slides were examined under an optical microscope (200 magnifications, Olympus, Japan). All procedures were performed according to the instructions provided by the kit.

For semi-quantitative analysis of α-SMA, more than 5 randomly selected fields (with 400 × magnification) per liver section of each mouse were recorded and scored by two independed pathologists according to intensity and proportion of α-SMA staining as previous report^26^. The score was evaluated as follows: The intensity score was determined as 0 (no staining), 1 (weak staining), 2 (moderate staining), and 3 (strong staining). The proportion score was determined as 1 (<30% of cells) and 2 (≥30% of cells). The intensity score and proportion score were multiplied together for a total score.

### Measurement of hepatic hydroxyproline content

The concentration of hepatic hydroxyproline from each mouse was determined with a commercial hydroxyproline testing kit (Jiancheng, Nanjing, China) according to the manufacturer’s instruction. In brief, frozen liver tissue (approximate 30 mg) was heated in 6 mM HCl at 120 °C for 4 h in sealed tubes. The samples were then centrifuged, and 10 ml of supernatant was mixed with 100 ml of chloramine-T solution (1.4% chloramine-T, 10% N-propanol, and 80% citrate-acetate buffer). This mixture was incubated for 20 min at room temperature. After that, Ehrlich’s solution was added, and the samples were incubated at 65 °C for 20 min. The absorbance was measured at 550 nm, and the concentration was determined by the standard curve created by cis-4-hydroxy-L-proline (Sigma-Aldrich, USA).

### Measurement of type I collagen content

The concentration of type I collagen from each mouse was detected using the mouse type I collagen testing kit (Jingmei, Yancheng, China) according to the manufacturer’s instruction. In brief, 50 μl of the sample (40 μl of sample diluent and 10 μl of hepatic homogenate) was accurately added to the plate and incubated at 37 °C for 30 minutes. The antibody was conjugated with HRP-labeled type I collagen antibody After thorough washing, the substrate TMB was added, TMB is converted to blue under the catalysis of HRP enzyme and converted to final yellow under the action of acid. The absorbance (OD) was measured at 450 nm using a microplate reader. The concentration of collagen type I in the sample was calculated by standard curve.

### RNAs isolation and quantitative real-time PCR

Total RNA was extracted from frozen tissues or cultured cells by using TRIzol reagent according to the manufacturer’s instructions (Invitrogen, Carlsbad, CA, USA). The RNA was reverse transcribed to complementary DNA. Quantitative real-time PCR was performed for by using SYBR green real-time PCR master mix according to the manufacturer’s instructions (Roche Applied Science, Mannheim, Germany). Sequences of the mouse and human gene-specific primers used in this study are listed in Table in Table S1. Relative quantification of each gene expression was calculated in terms of comparative cycling threshold (Ct) normalized by β-actin (Sangon, Shanghai, China) with 2^−ΔΔCt^.

### Western-blotting

Cellular proteins were isolated from hepatic tissues in RIPA lysis buffer (50 mM Tris-HCl, pH 7.4, 150 mM NaCl, 0.25% deoxycholic acid, 1% Nonidet P-40, 1 mM EDTA) (Millipore, Billerica, MA, USA) containing a protease inhibitor cocktail (Roche) and quantified with bicinchoninic acid protein concentration assay kit (Beyotime Biotech, Beijing, China). Protein of each sample was separated by electrophoresis in 10% SDS-PAGE with a Bio-Rad electrophoresis system (Hercules, CA, USA). After electrophoresis, the resolved protein was transferred onto PVDF membranes (Millipore). Membranes were then incubated with primary rabbit antibodies: α-SMA (1:1000, UCallM biotech Co., Ltd, Wuxi, China), MyD88 (1:1000, UCallM biotech Co., Ltd, Wuxi, China), p-NF-κB (1:1000, UCallM biotech Co., Ltd, Wuxi, China), TGF-β_1_ (1:1000, Affinity Biosciences, OH, USA), p-Smad2/3 (1:1000, Proteintech, Wuhan, China), BAMBI (1:1000, Proteintech, Wuhan, China) and β-actin (1:2000, Santa Cruz Biotechnology, CA, USA) at 4 °C overnight. The corresponding horseradish-peroxidase-conjugated secondary antibodies (anti-rabbit IgG, Santa Cruz Biotechnology, CA, USA. 1:2000 dilutions) were incubated for 2 h at room temperature. The protein bands were visualized using enhanced chemiluminescence reagents (Millipore). The optical density of each protein in each sample was normalized against β -actin.

### Statistical Analysis

The data are shown as mean values ± standard error of the mean (SEM) from at least three independent experiments. Differences between multiple groups were compared using one-way ANOVA with post-hoc Bonferroni correction. A Student’s *t*-test was used for comparisons between two groups. A *P*-value < 0.05 was considered significant.

## Results

### TLR4 mutation attenuates biliary fibrosis in *C*. *sinensis*-infected mice

**Figure 1:**
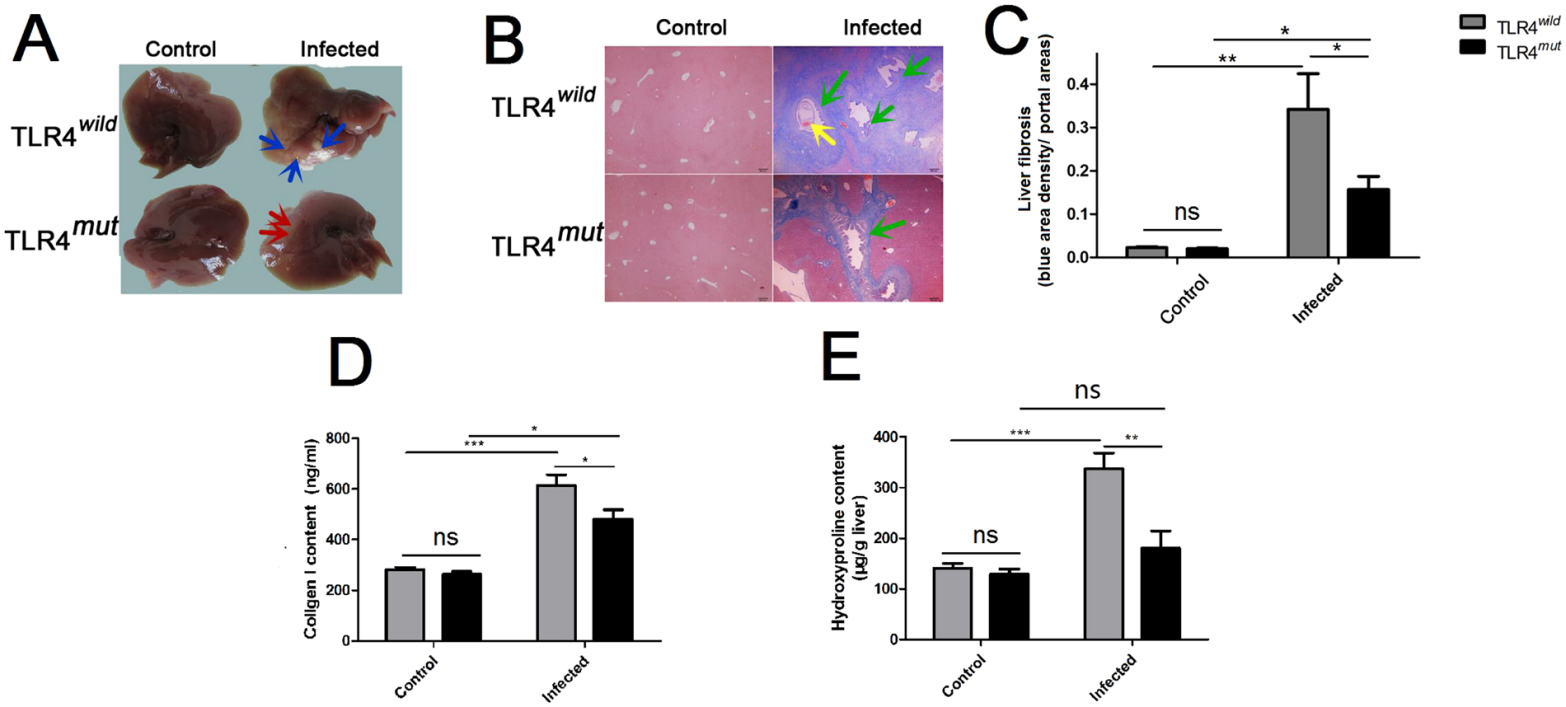
The mice with TLR4 mutation showed an attenuated pathogen-associated biliary fibrosis caused by *Clonorchis sinensis*. The mice with TLR4 wild and mutation were orally administrated by 45 metacercariae *C*. *sinensis* or PBS, respectively. These mice as well as healthy control mice were executed and livers were harvested on 28 days of *C*. *sinensis* post-infection. (**A**) Gross clinic hepatic changes of TLR4^*wild*^ and TLR4^*mut*^ C3H mice caused by *C*. *sinensis*. Blue arrows indicate massive nodules with different size in the hepatic lobes in the TLR4^*wild*^ mice infected by *C*. *sinensis*, and red arrows showed limited pale area on the surface of lobes without nodules in TLR4^*mut*^mice, compared with TLR4^*wild*^ mice. (**B**) Representative Masson staining slides showed collagen fiber deposition (blue stripes). Scale bars = 200 μm. Green arrows showed the proliferation of biliary epitheliums and enlarged the bile ducts in the mice of *C*. *sinensis*-infected mice, and the yellow arrow shows the worm’s body in the enlarged bile ducts. (**C**) The mean optical density of collagen fibers indicated by Masson staining was digitized and quantitated in the liver of healthy and *C*. *sinensis*-infected mice by on Image-Pro Plus software. (**D**) Concentrations of Collagen I in the hepatic homogenate of healthy control and *C*. *sinensis*-infected mice with TLR4^*wild*^ and TLR4^*mut*^. (**E**) Concentrations of hydroxyproline in the hepatic homogenate of healthy normal control and *C*. *sinensis*-infected mice with TLR4^*wild*^ and TLR4^*mut*^. The data were obtained from 3~5 mice of three-independent experiment. The values were expressed as mean ± SEM. compared with indicated group, *P < 0.05, **P < 0.01, ***P < 0.0001.

The mice with C3H background (C3H/HeN with TLR4 normal function and C3H/HeJ with deficient function of TLR4) were administrated by gastric perfusion of 45 *C*. *sinensis* metacercariae as previous described^11^. Worms’ eggs in the liver of mice infected by *C*. *sinensis* were all detected in both strain mice on 28 day post-infection (the infection rates of C3H/HeN and C3H/HeJ were both 100%), suggesting that the *C*. *sinensis* were successful to develop mature in this strain mouse and the levels of TLR4 were highly increased on days of 28 post-infection in *C*. *sinensis*-infected C3H mice, compared with health normal mice^11^. Based on these observations, to determine whether TLR4 involves in the PABF or not, we developed *C*. *sinensis* caused PABF using C3H background mice which could provide us comparable information about the role of TLR4 when compared with C3H/HeJ mice which carries a spontaneous mutation and loss the TLR4 function^27^. In the present study, we found that TLR4^*wild*^ mice infected by *C*. *sinensis* showed a significant swelling of lobes and massive nodules with different sizes (approximate 0.1 cm ~1 cm in diameter) on the surface of lobes (yellow arrows), but TLR4^*mut*^ mice only showed a limited pale area on the surface of lobes (green arrows, Fig. 1A). Masson’s trichrome staining showed that collagen depositions were significantly increased both in TLR4 wild (Fig. 1B and C, *P* < 0.01) and TLR4 mutation mice (Fig. 1B and C, *P* < 0.05) on day 28 post-infection (p.i.), compared with no-infected mice. The results also showed that collagen depositions were significantly higher in TLR4^*wild*^ mice than in those mice with TLR4 mutation when they were infected by *C*. *sinensis* (Fig. 1B and C, *P* < 0.05). Similarly, we also found significantly increases of I type collagen both in TLR4^*wild*^ and TLR4^*mut*^ *C*. *sinensis*-infected mice when compared with no-infected mice. However, TLR4^*wild*^ mice that were infected by *C*. *sinensis* exhibited an elevated expression of collagen I in the sera, compared with TLR4^*mut*^ mice (Fig. 1D, *P* < 0.05). To confirm that an inactive TLR4 can attenuate *C*. *sinensis*-associated biliary fibrosis, we also measured the hydroxyproline (Hyp) contents in the liver of mice. The data showed that the concentrations of Hyp were about 2 times reduced in TLR4^*mut*^ mice, compared to wild mice when they were infected by *C*. *sinensis* on day of 28 p.i. (Fig. 1E, *P* < 0.05). Thus, these results demonstrate that TLR4 contributes to *C*. *sinensis* caused pathogen-associated biliary fibrosis.

### TLR4-mediated signaling pathway is partly responsible for *C*. *sinensis*-associated biliary fibrosis

**Figure 2:**
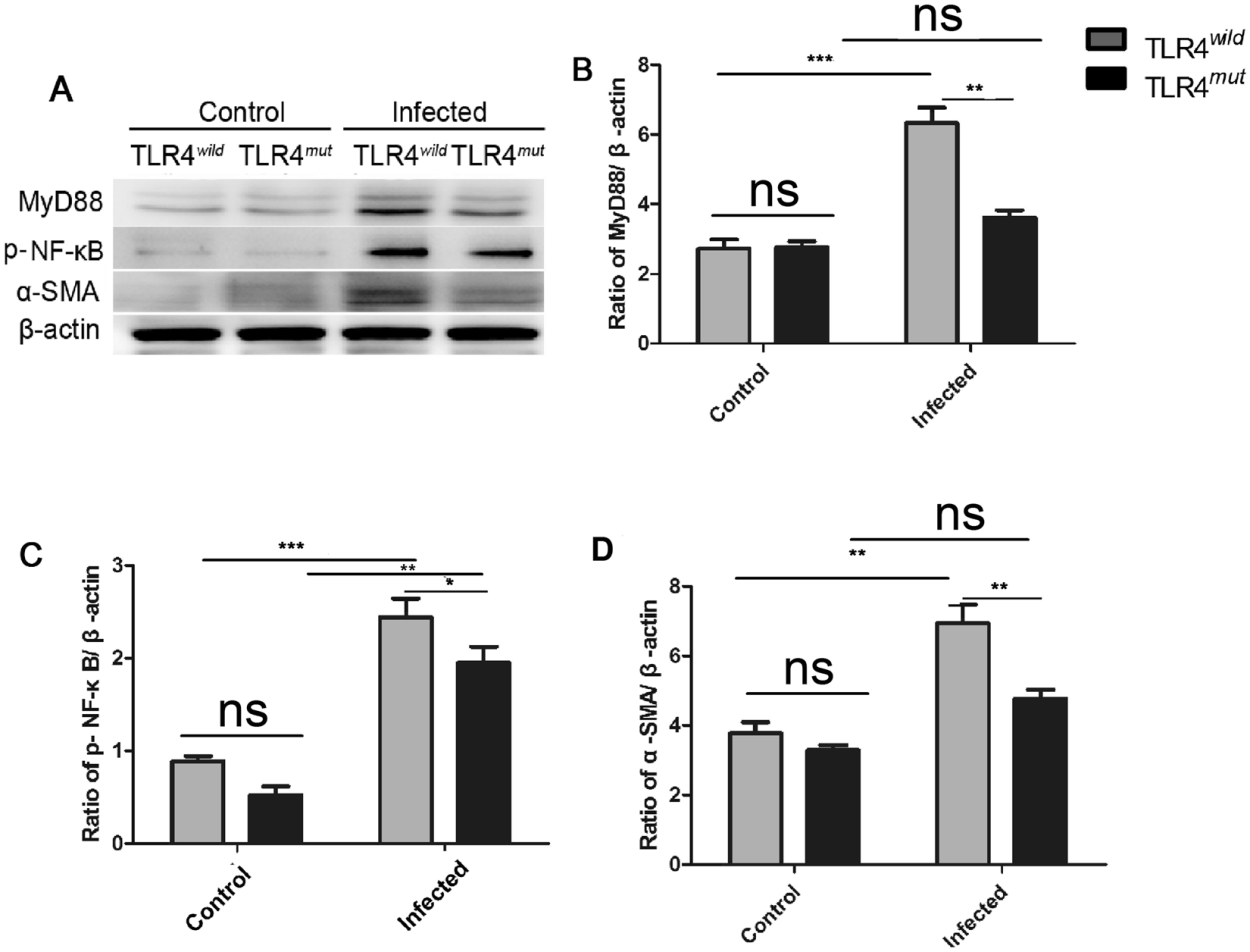
TLR4 signaling pathway is involved in the pathogen-associated biliary fibrosis caused by *Clonorchis sinensis*. Livers from TLR4 wild and mutated mice infected with or without *C*. *sinensis* were harvested, and the sections of tissue containing bile ducts were homogenated or fixed by 4% formalin solution. (**A–D**) Western-blot shows relative expression of MyD88 (**B**), p-NF-κB (**C**) and α-SMA (**D**) in the liver of healthy normal control and *C*. *sinensis*-infected mice with TLR4^*wild*^ and TLR4^*mut*^. The blots of each group were run under the same experimental conditions and the images were from the same gel. The data were obtained from 3~5 mice of three-independent experiment. The values were expressed as mean ± SEM. Compared with indicated mice, *P < 0.05, **P < 0.01,***P < 0.001.

**Figure 3:**
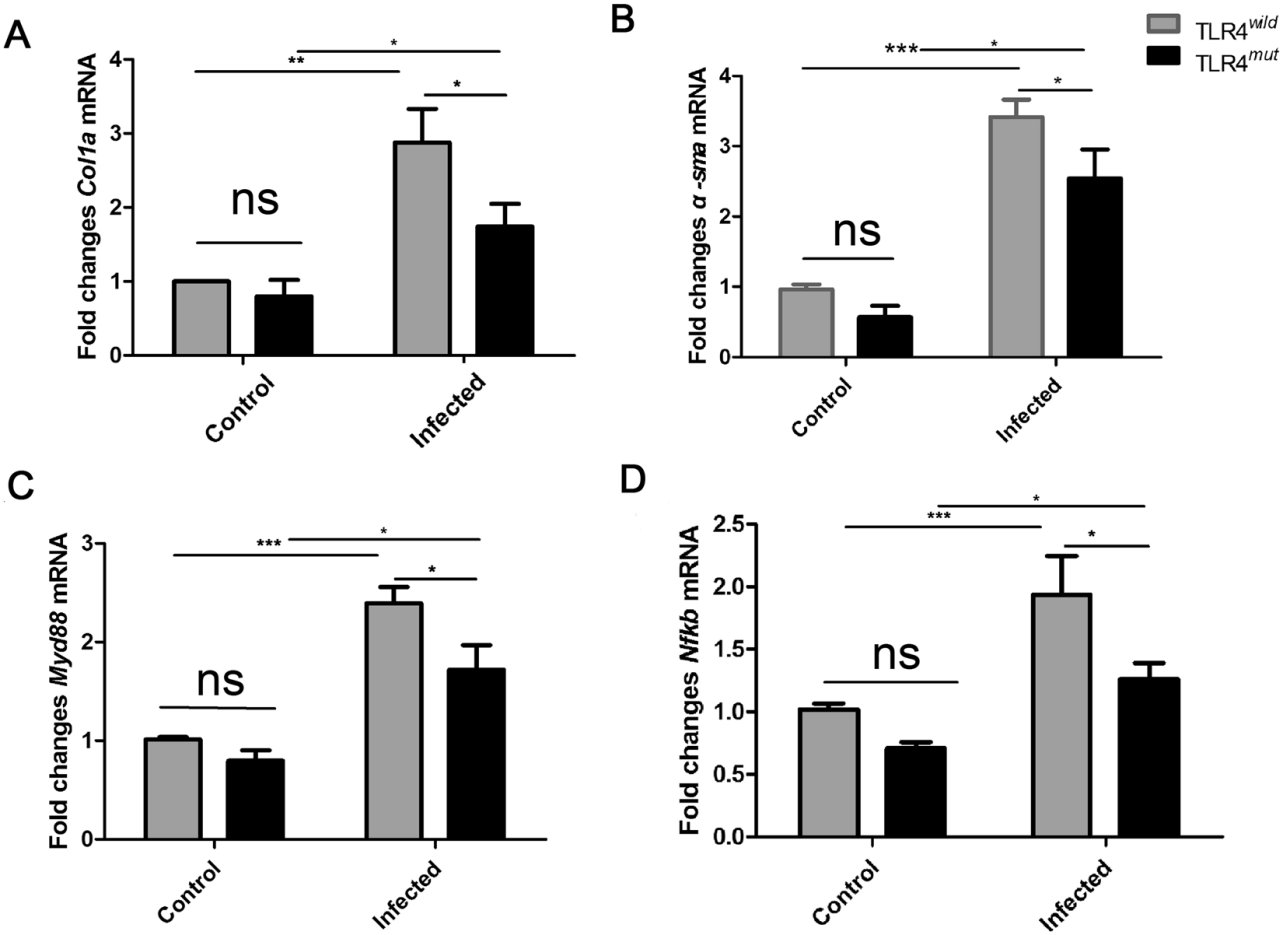
The relative expression of *Nfkb*, *Myd88*, *Col1a* and *α*-*sma* transcripts in the liver of TLR4 wild and mutated mice infected by with or without *C* *lonorchis* *sinensis*. The sections of tissue containing bile ducts were homogenated. Total RNAs were extracted and reverse transcribed to complementary DNA. Fold changes of *Col1a* (**A**), *α-sma* (**B**), *Myd88* (**C**) and *Nfkb* (**D**) transcripts to β-actin were measured by real-time qPCR. The data were obtained from 3~5 mice of three-independent experiment. The values were expressed as mean ± SEM. Compared with indicated mice *P < 0.05, **P < 0.01, ***P < 0.001.

We next determined whether TLR4-mediated signaling pathway was involved in *C*. *sinensis* pathogen-associated biliary fibrogenesis or not. We performed real-time PCR and Western blot analyses to determine differential expression of molecules known to be involved in TLR4 signaling pathway and the biomarkers of hepatic fibrosis, respectively. Firstly, compared with no-infected mice, *C*. *sinensis*-infected mice showed higher levels of α-SMA (determined both by western-blot and real-time PCR, Fig. 2D and Fig. 3B, *P* < 0.01) and I Collagen (determined by real-time PCR, Fig. 3A, *P* < 0.05) regardless of TLR4^*wild*^ or TLR4^*mut*^ mice. However, the level of α-SMA (determined by western-blot and real-time PCR, Fig. 2D and Fig. 3B, *P* < 0.01) or *Col1a* transcripts (determined by real-time PCR, Fig. 3A, *P* < 0.05) the liver of *C*. *sinensis*-infected WT mice were also significantly higher in than those from TLR4^*mut*^ mice.

For TLR4 signaling pathway, western-blot analyses showed that the levels of MyD88 and phosphorylated NF-κB were both significantly increased in the liver of TLR4^*wild*^ as well as TLR4^*mut*^ mice that were infected by *C*. *sinensis*, when compared with healthy control mice accordingly (Fig. 2A–C). However, in *C*. *sinensis*-infected TLR4^*mut*^ mice, the expression of MyD88 and p-NF-κB were significantly lower than those in TLR4^*wild*^ mice that infected by *C*. *sinensis* (Fig. 2A–C). The real-time PCR data also showed that the transcripts of *Nfkb* and *Myd88* were increased about 2 times in *C*. *sinensis*-infected TLR4 wild mice, compared with no-infected mice with the same type (Fig. 3C and D, *P* < 0.0001). Moreover, compared with *C*. *sinensis*-infected TLR4^*mut*^ mice, there were significantly increases in the expression of *Myd88* (Fig. 3C, *P* < 0.05) and *Nfkb* (Fig. 3D, *P* < 0.05) in *C*. *sinensis*-infected TLR4^*wild*^ mice. These data suggest that impairment of TLR4 signaling prevents the development of PABF caused by *C*. *sinensis*.

### Impairment of TLR4 prevents TGF-β mediated PABF caused by *C. sinensis*

**Figure 4:**
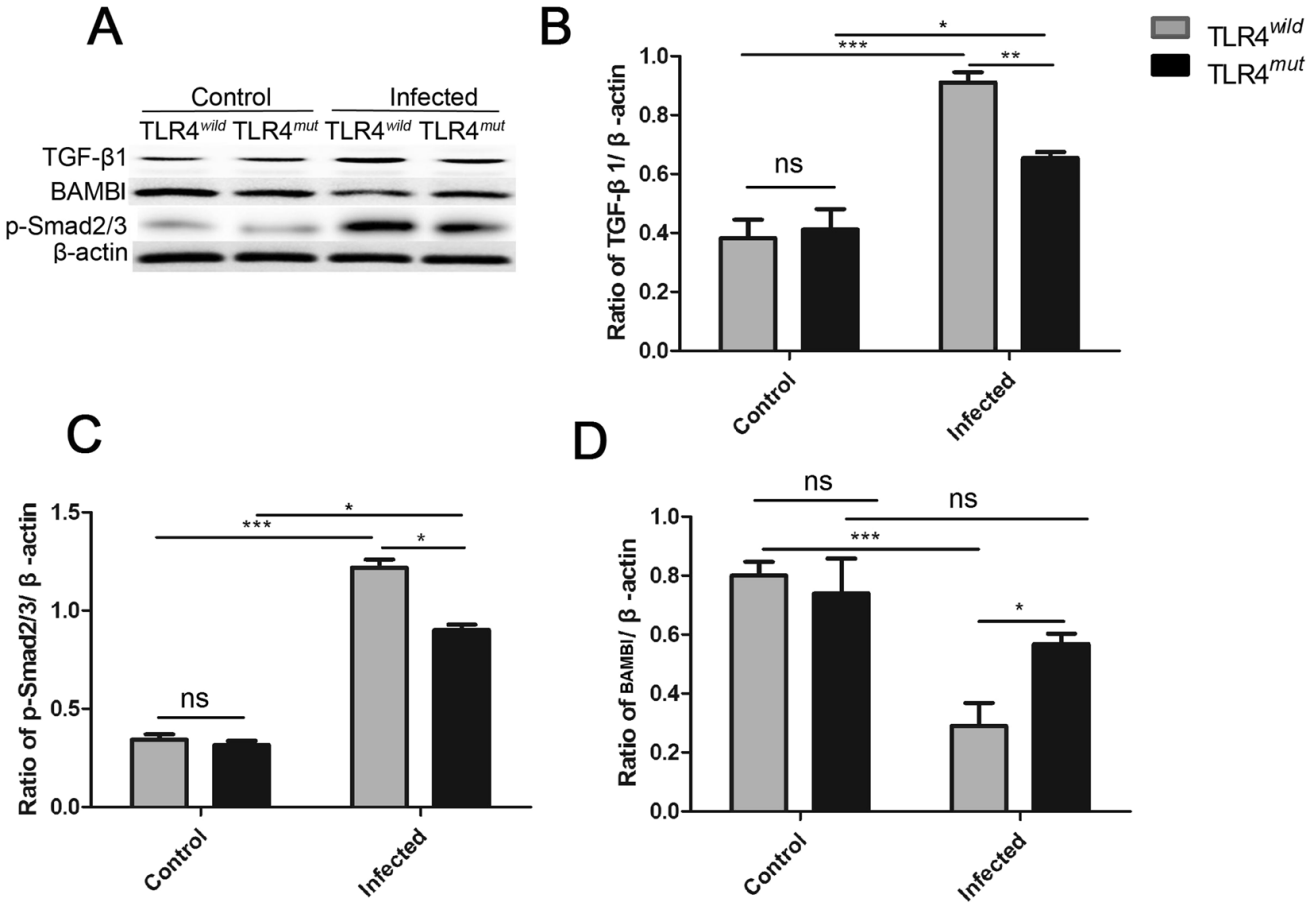
TLR4 orchestrates TGF-β/Smads signaling to promote pathogen-associated biliary fibrosis caused by *Clonorchis sinensis*. Livers from TLR4 wild and mutated mice infected with or without *C*. *sinensis* were harvested, and the sections of tissue containing bile ducts were homogenated for western-blot. (**A–D**)**:** the relative expression of TGF-β1 (**B**), p-Smad2/3 (**C**) and BAMBI (**D**) were analyzed in the liver of TLR4^*wild*^/TLR4^*mut*^ mice infected with or without *C*. *sinensis*-infected by Western blotting. The blots of each group were run under the same experimental conditions and the images were from the same gel. The data were obtained from 3~5 mice of three-independent experiment. The values were expressed as mean ± SEM. Compared with indicated group, *P < 0.05, **P < 0.01, ***P < 0.001.

As TGF-β is critical for HSC activation and ECM productions, and our previous study showed that TGF-β signaling pathway is activated in the mice infected by *C*. *sinensis* and promote the development of biliary fibrosis^25^, we further investigated the effect of TLR4 on the activation of TGF-β signaling pathway. Compared with normal control mice, the expression of TGF-β_1_ and p-Smad2/3 were significantly increased in both TLR4 wild and mutation type mice that were infected by *C*. *sinensis* (Fig. 4A, B and C). However, the mice with TLR4 mutation suppressed the expression of TGF-β_1_ and p-smad2/3, compared with the wild type mice exposed to *C*. *sinensis* (Fig. 4A, B and C). Furthermore, previous study showed that TLR4 ligands down-regulated the transforming growth factor (TGF)-β pseudoreceptor (BAMBI) to promote the sensitiveness of HSC to TGF-β_1_^22, 24^. Similarly, in the present study, we also observed that the level of BAMBI in the TLR4^*mut*^ mice was significantly increased compared with TLR4^*wild*^ mice when they were challenged to the same dose of *C*. *sinensis* metacercariae (Fig. 4D, *P* < 0.05). These data show that the deficiencies of TLR4 attenuates TGFβ mediated PABF caused by *C*. *sinensis*.

### The decreased activation of HSCs in the liver of TLR4^*mut*^ mice infected by *C*. *sinensis in situ*

**Figure 5:**
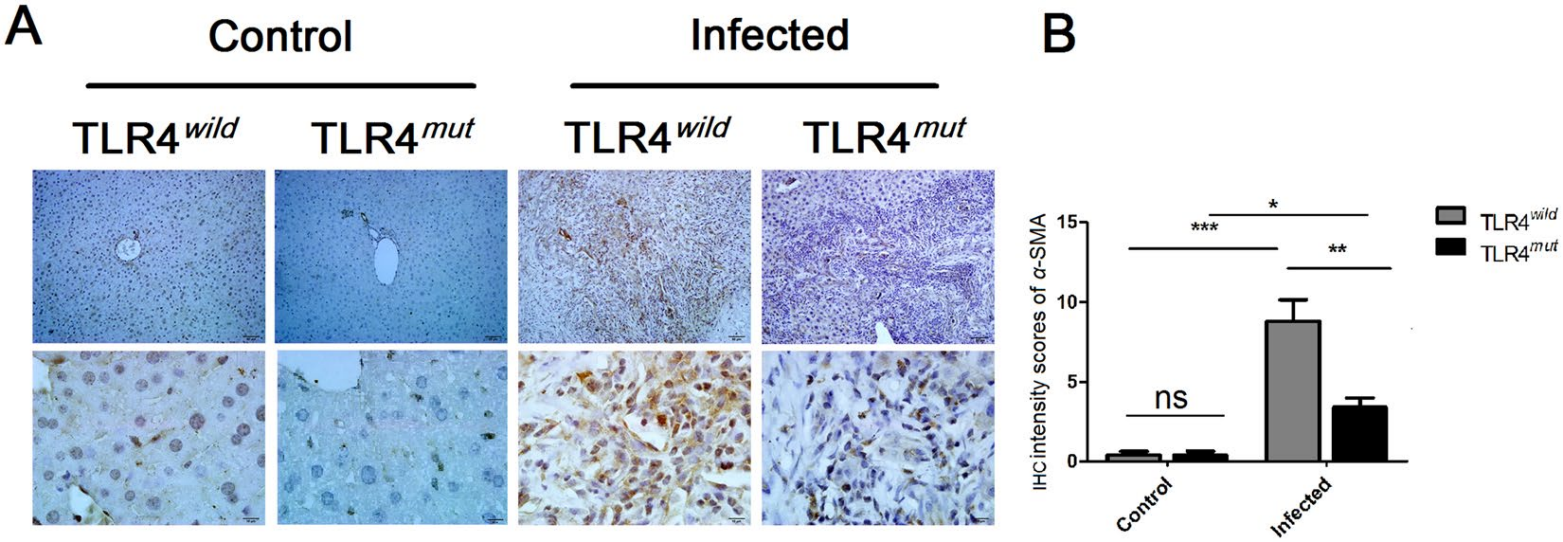
Impairment of TLR4 attenuates the activation of HSCs in the PABF caused by *Clonorchis sinensis*. Livers from TLR4 wild and mutated mice infected with or without *C*. *sinensis* were harvested, and the sections of tissue containing bile ducts were fixed by 4% formalin solution for IHC. The activation of HSCs was indicated by immunohistochemical staining of α-SMA. (**A**) Representative images of α-SMA staining on HSCs of TLR4 wild or mutated mice as indicated. Scale bars for upper panel are 50 μm and for the nether panel are 10 μm, respectively. (**B**) The expression of α-SMA were semi-quantitated by independent pathologists as described in Methods and Materials. The data were obtained from 3~5 mice of three-independent experiment. The values were expressed as mean ± SEM. Compared with indicated mice, *P < 0.05, **P < 0.01, ***P < 0.001.

As a biomarker of activated HSCs differentiate into myofibroblasts, α-SMA was stained to assess the activation of HSCs in mice of TLR4 wild and mutated mice infected by *C*. *sinensis in situ*. For no-infected mice, the α-SMA-expressed HSCs or myofibroblasts were hardly found in these mice including TLR4^*wild*^ and TLR4^*mut*^ healthy control mice (Fig. 5A), however, the abundant HSCs and myofibroblasts were stained strongly by anti-α-SMA antibody around bile ducts in *C*. *sinensis*-infected mice including TLR4^*wild*^ and TLR4^*mut*^ mice (Fig. 5A). However, the expression of α-SMA was significantly increased in the liver of *C*. *sinensis*-infected TLR4^*wild*^ mice, compared with TLR4^*mut*^ mice (Fig. 5B, *P* < 0.05), suggesting that the functional defect in TLR4 attenuates the activation of HSCs in the mice of PAPF caused by *C*. *sinensis*.

## Discussion

PABF is a particular liver fibrosis which is characterized by proliferation of biliary epithelial cells, portal/periportal infiltration of inflammatory cells and the activation of myofibroblasts^5^. However, the mechanisms underlying the persistence and progression of PABF remain poorly understood. Our present study shows that TLR4 deficiency ameliorates experimental biliary fibrosis caused by *C*. *sinensis in vivo* and the activation of ECMs-producing HSCs was attenuated when TLR4 signaling pathway was blocked *in vitro*. Taken together, our observations suggest that TLR4 promotes the PABF caused by *C*. *sinensis*.

Previous studies showed that the strain of C3H mouse developed a massive peribiliary fibrosis on 28 day post-infection and the junior *C*. *sinensis* develops into adult worm in the biliary ducts of C3H mice about on 21~23 day post-infection^11, 28^. In addition, the eggs of worms in the liver of the *C*. *sinensis*-infected mice were all detected in the present study (with the 100% infection rates regardless of TLR4mut or TLR4 wild mice). These results also suggest that C3H mouse that showed the similar characterizations for human clonorchiasis is an appropriate model for *C*. *sinensis*-caused biliary fibrosis. Importantly, our previous studies also showed that the expression of TLR4 was increased and TLR4 was highly expressed in HSCs and myoblasts cells in *C*. *sinensis*-infected C3H mice on 28 day of post-infection, which suggests that TLR4 probably plays a role in the process of this type of liver fibrosis^11^. Therefore, to further elucidate the underlying mechanisms of TLR4 in the biliary fibrosis caused by *C*. *sinensis*, we employed the C3H/HeJ mouse that carries a mutation of TLR4 gene and leads to the functional deficiency of TLR4 to compare with C3H/HeN *in vivo* ^27^. The data from these two types of *C*. *sinensis*-infected mice allowed us to analyze the roles of TLR4 in the peribiliary fibrosis caused by *C*. *sinensis*.

There are mounting evidences showed that innate immune responses participate in the fibrogenesis caused by various insults and injuries^22, 29–31^. TLRs are a conserve family of PPRs which initial innate responses and adaptive immune responses in infectious and no-infectious diseases^32^. However, TLR4 appears to play diverse roles in the regulation of fibrosis under different microenvironments^33^. For example, it was shown that TLR4 signaling pathway promotes the activations of HSCs and contributes to liver fibrosis through coordination of TGF-β signaling in the liver fibrosis of no-infectious disease^22, 34^ while TLR4 as a negative regulator prevents fibrosis in the models of lung injuries^35, 36^. Recent study showed that HSCs are the universal contributor to collagen-producing myofibroblasts in models of toxic, cholesteric and fatty liver fibrosis^16^, which support our hypothesis that TLR4 mutation mice were protected from *C*. *sinensis*-caused biliary fibrosis and the increased activation of the HSCs contributed to hepatic fibrogenesis caused by *C*. *sinensis* in TLR4^*wild*^ mice.

TGF-β is the most powerful mediator and play a central role in the activation of HSCs that mediates fibrosis and our previous studies showed that this pathway also involved in the *C*. *sinensis*-caused fibrosis^25^. But it seems that TGF-β alone is not sufficient to develop liver fibrosis in *Tgfb* gene transgenic mice as it requires synergistic molecules to induce fibrosis^2^. In the present study, we found that the expression of TGF-β pseudoreceptor, BAMIB were significantly decreased in the liver of TLR4 wild mice infected by *C*. *sinensis* as well as in the HSCs treated with ESPs of *C*. *sinen*sis. It has been proposed that Bambi is co-expressed on the surface of HSC and the down-regulated BAMBI is responsible for the TGF-β-mediated HSC activation^22, 37, 38^. We found that the expression of BAMBI was significantly increased and the downstream pSmad2/3 was decreased when the function of TLR4 was blocked. These data are in line with previous studies, which also showed that TLR4-mediated TNF-α depresses BAMBI transcriptional activity independent on NF-κB^39^. Our previous study showed that the expression of TNF-α was significantly decreased in primary biliary epithelial cells (BECs) stimulated by ESPs when TLR4 signaling pathway was blocked^40^. Therefore, it may be conceivable that the decreased TNF-α due to a defect in TLR4 signaling pathway was probably responsible for the down-regulated the expression of BAMBI and then consequently make a negatively effect on the activation of HSCs as well as the process of biliary fibrosis caused by *C*. *sinensis*.

## Conclusion

In conclusion, to our knowledge, the findings of the present study reported provide first evidence to demonstrate the roles of TLR4 in *C*. *sinensis* caused PABF. The data could contribute to a better understanding of innate immune responses in the development of PABF. Given the participation of TLR4 in the *C*. *sinensis*-caused PABF, strategies to inhibit TLR4 signaling should be explored.

## Electronic supplementary material

Supplementary Information 
