# Supplementary material for: The roles of Toll-like receptor 4 in the pathogenesis of pathogen-associated biliary fibrosis caused by Clonorchis sinensis
Source: Sci Rep. 2017 Jun 20;7:3909. doi: 10.1038/s41598-017-04018-8 (PMC5478609; doi:10.1038/s41598-017-04018-8)

**The roles of Toll-like receptor 4 in the pathogenesis of pathogen-associated biliary** **fibrosis caused by *Clonorchis sinensis***

**Chao Yan^1,＋^, Bo Li^1,2＋^, Fang Fan^1^, Rui Ma^1^, Ying Du^1^, Xiao-Dan Chen^1^, Xiang-Yang Li^1^, Bo Zhang^1^, Qian Yu^1^, Yu-Gang Wang^1^, Ren-Xian Tang^1**^, Kui-Yang Zheng^1*^**

*Corresponding author:

E-mail: [ZKY02@163.com](mailto:ZKY02@163.com) (KYZ)

**Co-corresponding author:

E-mail: [Tangrenxian-t@163.com](mailto:Tangrenxian-t@163.com) (RXT)

^+^These authors contributed equally to this work.

**Supplementary information**

**Table S1 the primers used in this study.**

| **Gene name** |  | **Sequences** |
| --- | --- | --- |
| m*a-Sma* | Forward | 5′-AAGAGCATCCGACACTGCTGAC-3′ |
|  | Reverse | 5′-AATAGCCACGCTCAGTCAGG-3′ |
| m*Col1A1* | Forward | 5′-CAGGGTATTGCTGGACAACGTG-3′ |
|  | Reverse | 5′-GGACCTTGTTTGCCAGGTTCA-3′ |
| m*Tgfb* | Forward | 5′-GTGTGGAGCAACATGTGGAACTCTA-3′ |
|  | Reverse | 5′-TTGGTTCAGCCACTGCCGTA-3′ |
| m*Tlr4* | Forward | 5´-TGA CAG GAA ACC CTA TCC AGA GTT-3´ |
|  | Reverse | 5´-TCT CCA CAG CCA CCA GAT TCT-3´ |
| m*Myd88* | Forward | 5′-AAG AAA GTG AGT CTC CCC TC-3′ |
|  | Reverse | 5′-TCC CAT GAA ACC TCT AAC AC-3′ |
| m*Nfkb* | Forward | 5′-AGCACAGATACCACCAAGAC-3′ |
|  | Reverse | 5′-TCAGCCTCATAGTAGCCATC-3′ |
| m*β-actin* | Forward | 5′- AACTCCATCATGAAGTGTGA -3′ |
|  | Reverse | 5′- ACTCCTGCTTGCTGATCCAC-3′ |
| hTlr4 | Forward | 5′- TGGAAGTTGAACGAATGGAATGTG-3′ |
|  | Reverse | 5′-ACCAGAACTGCTACAACAGATACT-3′ |
| h*a-Sma* | Forward | 5′- TTCATCGGGATGGAGTCTGCTGG-3′ |
|  | Reverse | 5′-TCGGTCGGCAATGCCAGGGT-3′ |
| h*Col1A1* | Forward | 5′- ACTGGTGAGACCTGCGTGTA-3′ |
|  | Reverse | 5′- AATCCATCGGTCATGCTCTC-3′ |
| h*Tgfb* | Forward | 5′-GCAACAATTCCTGGCGATAC-3′ |
|  | Reverse | 5′-CTAAGGCGAAAGCCCTCAAT-3′ |
| h*Myd88* | Forward | 5′-GCAGAGCAAGGAATGTGACT-3′ |
|  | Reverse | 5′-CGCAGACAGTGATGAACCTC-3′ |
| h*Nfkb* | Forward | 5′-CTGAGTCCTGCTCCTTCCAA-3′ |
|  | Reverse | 5′-CGGTGTAGCCCATTTGTCTC-3′ |
| h*β-actin* | Forward | 5′-CATGTACGTTGCTATCCAGGC-3′ |
|  | Reverse | 5′-CTCCTTAATGTCACGCACGA -3′ |

**Figure S1.** **Full-length gel images for western blotting results.** Full-length blot images for Figure 2A and Figure 4A.


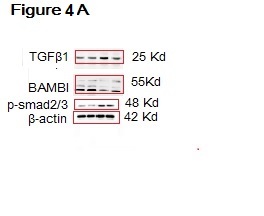

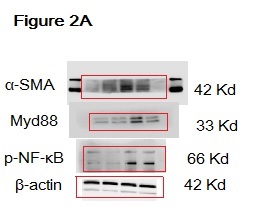

Supplement: Supplementary file 1 — Supplementary Information [file 41598_2017_4018_MOESM1_ESM.doc]
